# Precision Diagnostics by Affinity-Mass Spectrometry: A Novel Approach for Fetal Growth Restriction Screening during Pregnancy

**DOI:** 10.3390/jcm9051374

**Published:** 2020-05-07

**Authors:** Charles A. Okai, Manuela Russ, Manja Wölter, Kristin Andresen, Werner Rath, Michael O. Glocker, Ulrich Pecks

**Affiliations:** 1 Proteome Center Rostock, University Medicine and Natural Science Faculty, University of Rostock, 18051 Rostock, Germany; charles.okai@uni-rostock.de (C.A.O.); manuela.russ@med.uni-rostock.de (M.R.); manja.woelter@googlemail.com (M.W.); 2 Department of Obstetrics and Gynecology, Medical Faculty, University of Rostock, Clinic Südstadt, 18059 Rostock, Germany; 3 Department of Obstetrics and Gynecology, Medical Faculty, University of Schleswig-Holstein, Campus Kiel, 24105 Kiel, Germany; Kristin.Andresen@uksh.de (K.A.); wrath@ukaachen.de (W.R.); 4 Department of Obstetrics and Gynecology, Medical Faculty, RWTH Aachen University, 52062 Aachen, Germany

**Keywords:** FGR, SGA, proteome profiling, affinity-MS, pregnancy complications, apolipoproteins

## Abstract

Fetal growth restriction (FGR) affects about 3% to 8% of pregnancies, leading to higher perinatal mortality and morbidity. Current strategies for detecting fetal growth impairment are based on ultrasound inspections. However, antenatal detection rates are insufficient and critical in countries with substandard care. To overcome difficulties with detection and to better discriminate between high risk FGR and low risk small for gestational age (SGA) fetuses, we investigated the suitability of risk assessment based on the analysis of a recently developed proteome profile derived from maternal serum in different study groups. Maternal serum, collected at around 31 weeks of gestation, was analyzed in 30 FGR, 15 SGA, and 30 control (CTRL) pregnant women who delivered between 31 and 40 weeks of gestation. From the 75 pregnant women of this study, 2 were excluded because of deficient raw data and 2 patients could not be grouped due to indeterminate results. Consistency between proteome profile and sonography results was obtained for 59 patients (26 true positive and 33 true negative). Of the proteome profiling 12 contrarious grouped individuals, 3 were false negative and 9 were false positive cases with respect to ultrasound data. Both true positive and false positive grouping transfer the respective patients to closer surveillance and thorough pregnancy management. Accuracy of the test is considered high with an area-under-curve value of 0.88 in receiver-operator-characteristics analysis. Proteome profiling by affinity-mass spectrometry during pregnancy provides a reliable method for risk assessment of impaired development in fetuses and consumes just minute volumes of maternal peripheral blood. In addition to clinical testing proteome profiling by affinity-mass spectrometry may improve risk assessment, referring pregnant women to specialists early, thereby improving perinatal outcomes.

## 1. Introduction

Fetal Growth Restriction (FGR) is a pregnancy condition in which the fetus does not reach its genetically given growth potential. It is a major cause of fetal and neonatal morbidity and mortality, affecting about 3% to 8% of all pregnancies [1,2,3]. Clinically, FGR needs to be distinguished from constitutionally small for gestational age (SGA) fetuses, which represent “physiological smallness” and hence, are not of the same clinical concern. The current standard of detection of FGR and differentiation from SGA is based on ultrasound examinations [1,3,4]. Once identified, FGR pregnancies need intense observation and should be transferred to perinatal specialists to enhance surveillance and if necessary, induce labor to avoid intra-uterine death while balancing against the risk of prematurity.

Antenatal diagnosis has proven to reduce adverse perinatal outcomes and allows for proper and timely referral of the neonate to intensive care [5,6,7]. However, antenatal detection rates still are sparse and range at about 20–50%, even in high-income countries [8,9]. FGR diagnosis is often made by observation of fetal growth velocity, which can only be confirmed with significant delay in serial ultrasound measurements which are usually timed at least two weeks apart. Most important, the need for ultrasound equipment and highly trained ultrasound specialists limits routine screening for FGR/SGA in pregnant women in many countries, especially in low-income countries [2]. In recent studies, maternal serological biomarkers have been suggested to improve FGR detection rates. Among other biomarkers, soluble Fms-like thyrosinkinase-1 (sFlt-1) and placental growth factor (PlGF) were applied in concert with ultrasound biometry and maternal risk factor estimations to predict FGR outcome [10], indicating that molecular diagnosis improved clinical screening results.

In our recently published proteome profiling studies, using affinity-mass spectrometry with serum from cord blood as well as from maternal peripheral venous blood, we identified apolipoprotein C-II and apolipoprotein CIII protein species as potential candidates from neonates and from pregnant women to differentiate between FGR and CTRL. By use of five candidate proteins, we developed a proteome-based scoring system for the detection of FGR with high confidence [7,11]. However, owing to the aim of our former study to analyze changes in maternal and fetal blood in parallel, the control (CTRL) cohort contained individuals who gave birth prematurely for other reasons than FGR to match the FGR cohort for gestational age. This cohort matching limited interpretation and generalization of the data. 

In the present study, we aim at validating in different clinical scenarios the FGR-specific affinity-mass spectrometry-based serum proteome profiling procedure, which we developed [7,11,12,13,14]. For this purpose, we challenged the FGR proteome profile by supplementation of our previous cohorts (FGR II and CTRL II) with three further cohorts: 15 patients with severe early onset FGR requiring early delivery before 34 weeks of gestation (FGR I), 15 individuals with uncomplicated pregnancies who gave birth near term (CTRL I) but blood was sampled at similar gestational ages as with cohort FGR I, and 15 donors with otherwise uncomplicated pregnancies, i.e., without features of FGR, classified as SGA by antenatal sonography (SGA I).

## 2. Materials and Methods

### 2.1. Patient and Control Individual Cohorts

The study was approved by the Ethics Committee of the Rheinisch-Westfälisch Technische Hochschule (RWTH) Aachen, Germany (EK 138/06, EK 119/08, EK 154/11). Written informed consent was obtained from all participating women. At time of inclusion in the study, sonographic examinations were done to classify patients into study cohorts using Logiq 5 or Voluson 730 Expert Ultrasound Systems (GE Healthcare Systems, Solingen, Germany). The regression equation including biparietal diameter, femur length, as well as head and abdominal circumferences, proposed by Hadlock et al. [15], was used to estimate fetal weight. Fetal and neonatal birth weight percentiles were determined according to the population-based newborn weight charts, as described previously [16]. FGR was defined in accordance with national and international guidelines [3], as described earlier [7,11,12,13,16]. In addition to having an estimated fetal weight below the 10th percentile, one of the following criteria had to be fulfilled: (i) deceleration of fetal growth velocity during the last 4 weeks, (ii) elevated resistance index in umbilical artery Doppler sonography above the 95th percentile or absent or reversed end-diastolic blood flow (ARED), (iii) fetal asymmetry (head to abdominal circumference ratio above the 95th percentile), or (iv) oligohydramnios (amniotic fluid index <5 cm). Neonatal weight was assessed post-partum to verify the diagnosis (<10th percentile). Healthy pregnant women with estimated antenatal fetal weight below the 10th percentile and confirmed fetal growth along their percentiles for more than 4 weeks and otherwise normal sonographic findings were classified into the SGA group. Patients with one of the following criteria were excluded from the trial: multiple gestation, fetal anomalies, abnormal fetal karyotype, patients with clinical or biochemical signs of infection, positive TORCH (Toxoplasmosis, Other (syphilis, varicella-zoster, parvovirus B19), Rubella, Cytomegalovirus, and Herpes) screening results, maternal diabetes mellitus/gestational diabetes, other severe maternal metabolic disorders, and patients’ withdrawal from the study, as was done previously [7,11,12,13,17]. The CTRL groups were chosen to best match to the FGR groups for clinical parameters, such as gestational age at blood sampling, BMI, parity, smoking status, and fetal sex. A part of the CTRL group, cohort CTRL II (patient numbers 151–165), *n* = 15, and a part of the FGR group, cohort FGR II (patient numbers 351–365), *n* = 15), have already been analyzed previously [7] and were included again for further developing the method. Then, 45 serum samples from other individuals were added to validate the established FGR proteome profile. Of those, 15 were from individuals with uncomplicated pregnancies with an estimated fetal weight adequate for gestational age; these were referred to as the CTRL I cohort (patient numbers 101–115). Another 15 blood samples were from patients with otherwise uncomplicated pregnancies carrying SGA fetuses; these were referred to as the SGA I cohort (patient numbers 201–215). The third group of 15 individuals with pregnancies with confirmed FGR fetus are referred to as the FGR I cohort (patient numbers 301–315) (Table 1 and Supplemental Table S1).

### 2.2. Blood Collection, Generation, and Storage of Peripheral Blood Serum

Blood was taken at admission to Hospital without considering special fasting status. Gestational age was calculated from the time point of the last menstrual period and was verified by first trimester ultrasound scan documentation, which is offered routinely in the German Health System between the 10th and the 14th week of gestation (Table 1). Blood samples (up to 9 mL each) were taken antenatally from each individual from the right or left cubital vein using monovette syringes (Serum Z/9 mL; Monovette^®^, Sarstedt, Germany). After incubation at room temperature for 15–30 min, samples were subjected to sedimentation of blood cells by centrifugation (Labofuge 400R, Fa. Heraeus Instruments, Waltham, MA, USA) at 2000 × g and at room temperature for 15 min [7,11,12,13,14,17]. Serum was aspirated, divided into aliquots (100 µL each), and stored at −80 °C. Altogether, time between blood sample collection and storage of frozen serum aliquots averaged around less than 1 h. Frozen serum aliquots were shipped on dry ice to the Proteome Center Rostock.

### 2.3. Protein Extract Preparation from Peripheral Blood Serum

Serum protein solutions were prepared from frozen serum samples according to established protocols [7,11,12,13,14]. In brief, from each thawed serum aliquot, 5 µL were incubated with 10 µL MB-HIC8 “binding buffer” and 5 µL of MB-HIC8 bead slurry for 1 min (Profiling Kit 100 MB-HIC8; Bruker Daltonik, Bremen, Germany). After washing the beads three times with 100 µL of “wash buffer” each, proteins were eluted with 10 µL of “elution buffer”, consisting of a 50% ACN solution. The magnetic MB-HIC8 beads with their hydrophobic surfaces enriched apolipoproteins and depleted the most abundant serum proteins, albumin and IgG [7,11,12,13].

### 2.4. MALDI-ToF MS Profiling of Serum Proteins and Internal Calibration of Mass Spectra

**Scheme 1:**
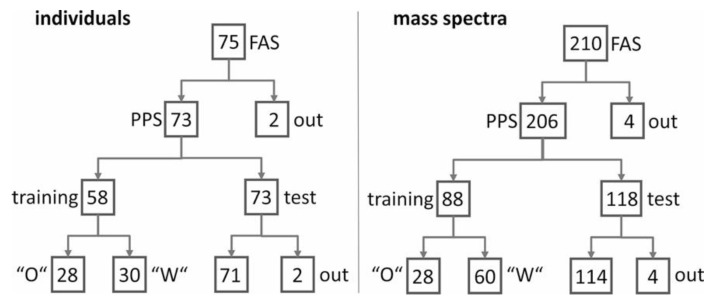
The use of individual samples and respective mass spectra. Mass spectra which were applied for training sets were not applied in test set analyses. FAS: full analysis set, PPS: per protocol set, “O“: training set “O”, “W“: training set “W”, out: individuals or mass spectra that were excluded, test: test set.

After extraction from the beads, serum protein-containing solutions (0.5 µL each) were spotted directly onto stainless steel MTP 384 target plates (Bruker Daltonik, Bremen, Germany) together with 0.5 µL ferulic acid solution (10 mg ferulic acid, SigmaAldrich, München, Germany) dissolved in 330 µL ACN/0.1% aqueous TFA (33/67, v/v) as matrix. After drying, 0.5 µL ferulic acid solution was added to each sample spot again and was allowed to dry, as was done previously [7,11,12,13]. Protein solutions were spotted in duplicate for each patient/donor for recording the first independent set of measurement series MS1 and the second independent set of measurement series MS2 of the same protein extract preparation (Supplemental Table S2). Protein mixtures embedded in the crystallized matrix were analyzed with a Reflex III MALDI TOF mass spectrometer (Bruker Daltonik, Bremen, Germany), which was equipped with a SCOUT source for delayed extraction and was operated in linear positive ion mode using an acceleration voltage of 20 kV. Spectra were recorded in a mass range from 4 to 20 kDa, respectively, accumulating 900 shots per spectrum. Spectra were externally calibrated using a commercially available Protein Calibration Standard (Bruker Daltonik, Bremen, Germany). All mass spectra were internally recalibrated using average masses of ion signals at m/z 6631.6 (singly charged and unmodified apolipoprotein C-I, Uniprot accession number P02654) and m/z 13,762.4 (singly charged and unmodified transthyretin, Uniprot accession number P02766). Ion signal areas were determined with the ClinProTools^™^ 3.0 software (Bruker Daltronik, Bremen, Germany) using the parameters as described previously [7,11,12,13,14,18]. Independent measurement series MS1 and MS2 were recorded for protein samples from individuals belonging to cohorts CTRL I, SGA I, and FGR I (in total, 90 mass spectra (Supplemental Table S2)). From individuals belonging to cohorts CTRL II and FGR II, we recorded four measurement series, MS1, MS2, MS3, and MS4, as described previously [7]. In total, from 75 individuals, 210 mass spectra were recorded, to which is referred to as the “full analysis set (FAS)” (Scheme 1 and Supplemental Scheme S1).

The mass spectra of the serum proteins from patient 115 contained very strong ion signals at m/z 11,527.0 and m/z 11,683.5, corresponding to the protein serum amyloid A1 minus the N-terminal arginyl residue and to full-length serum amyloid A1, respectively [19,20,21]. These ion signals were absent in all other mass spectra, indicating that this individual’s blood protein composition was different from all the others, which was the reason for exclusion. On the other hand, the mass spectra of the serum proteins from patient 315 could not be mass calibrated by the analysis software, although they resembled those of the other patients quite well with respect to ion signal abundances and rough locations of ion signal groups (data not shown). Nevertheless, the mass spectra were excluded as well (Scheme 1 and Supplemental Scheme S1), leaving a “per protocol set (PPS)” of 73 individuals (206 mass spectra).

### 2.5. Raw Data Processing and Formation of Quotients from Ion Signal Areas

After having determined the areas under each ion signal for each mass spectrum, we applied our established multi-parametric analysis procedure [7,11,18] in which the signal areas of five ion signals, those at m/z 8205, m/z 8766, m/z 8916, m/z 9422, and m/z 9713 (Supplemental Table S2), within each spectrum were brought into context to each other by forming quotients of ion signal areas. The signal area of the ion at m/z 8916 was divided by the signal area of the ion at m/z 8205 from one and the same spectrum to produce a value for spectra assessment (quotient A) as the first assessment value. The signal area of the ion at m/z 8766 over the sum of the signal areas of the ions at m/z 9422 plus m/z 9713 were determined (quotient B) as the second assessment value. The signal area of the ion at m/z 8916 over the sum of the signal areas of the ions at m/z 8766 plus m/z 9422 plus m/z 9713 were determined (quotient C) as the third assessment value. This data processing procedure was applied individually for each of the spectra.

### 2.6. Youden Index Analyses for Determining Cut-Off Values

To determine “best cut-off” values for quotients A, B, and C (see Raw Data Processing) that can be used to classify an individual spectrum (patient) as either belonging to the FGR I or the CTRL I group, a Youden index analysis was performed [17,22,23]. The first independent measurement series MS1 from both the per protocol set (PPS) for CTRL I (*n* = 14) and FGR I (*n* = 14) were chosen as training set “O” (Scheme 1 and Supplemental Scheme S1). The procedure is explained with quotient A as an example. First, all 28 patient spectra from the PPS were ranked according to their quotient values (Supplemental Table S3). Then, two theoretical quotient A values were added. The first theoretical quotient A value was determined by subtracting the value “1” from the lowest quotient A value and the second by adding the value “1” to the highest quotient A value. These two additional quotient A values were added at the top and the bottom of the quotient A list, respectively. Next, linear interpolation [24] between each pair of two neighboring concentration values was used to determine the “test cut-off” values and with each “test cut-off” value, it was assessed how many of the samples had quotient A values below this “test cut-off” value and how many had quotient A values above that value. Next, it was determined which of the samples were true positives (TP) and which were false positives (FP) by labeling spectra according to ultrasound assessment data (the "gold standard"). The sensitivity and specificity [25] were calculated for each “test cut-off” point. In addition, at each “test cut-off” value, the Youden index (J = sensitivity + specificity − 1) was determined [17,22,23]. The highest J value (Jmax) in the list of samples determined the best discrimination threshold for the quotient A values, i.e., the “best cut-off” value within the samples within this data set. This procedure was repeated for quotient B and quotient C values accordingly using data from the training set “O” (Supplemental Tables S4 and S5).

### 2.7. Cumulative Score Assignment

The “cut-off” values of 4.2, 5.0, and 4.0 for quotients A, B, and C, respectively, obtained from training set “O” were combined with the “cut-off” values of 3.4, 7.0, and 5.1 for quotients A, B, and C, respectively, from our previous study (training set “W”) [7]. These combined “cut-off” values were then applied to the “validation” test set, which consisted of a second independent measurement series MS2 of the same CTRL I and FGR I sera plus the third and fourth measurement series, MS3 and MS4, of CTRL II and FGR II sera [7]. The following scoring rules were applied: when the quotient value of a specific spectrum (sample) was below or equal to the lower of the two “cut-off” values, a score of “0.0” was assigned. When the quotient value was above the lower of the two “cut-off” values but below or equal to the upper of the two “cut-off” values, a score of “0.5” was assigned. When the quotient value was above the upper of the two “cut-off” values, a score of “1.0” was assigned. This weighting procedure was applied independently to each of the three ion signal ratios A, B, and C for test set “development”. Then, the score values of each spectrum from all three ion signal ratios were summed up so that each spectrum (each sample) reached a cumulative score between “0.0” and “3.0”.

### 2.8. Bioinformatic and Biostatistical Analysis

Clinical and biometric data analysis was carried out using the “statistical analysis software, SAS”, version 9.1 (SAS Institute, Cary, NC, USA). Clinical data were evaluated by two-way ANOVA analysis of variance and expressed as mean and 95% confidence interval. Differences of serum parameters were tested for significance using the Mann-Whitney *U* test association analyses and Spearman’s rank correlation (rho). Graphical representations, such as box-and-whisker plots [26], sensitivity, specificity, and area under the curve from the receiver operator characteristic (ROC) analysis, [27,28] were done using the Origin software (version. 8.1 G; OriginLab Corporation, Northampton, MA; USA). Quotient values A, B, and C of protein ion signal areas were graphically represented in heat maps. Hierarchical clustering was performed based on the complete linkage method and Spearman’s correlation coefficient as a measure of similarity. Signal intensities were centered and scaled row-wise for visualization purposes [7,13,29]. Unsupervised principle components analysis (PCA) was performed with quotient values A, B, and C of protein ion signal areas using MATLAB ver. 9.5.0 (R2018b), The MathWorks^®^, Inc., Natick, MA, USA [29,30]. The first two PCs were selected to project the data into a subspace, which is useful for visualization using the Origin software, and as input for a support vector machine algorithm (SVM). SVM was used to calculate the separation line for the classifier based on PCA projection.

### 2.9. Power Analysis

A power analysis was carried out [7,12,13,14] to evaluate the minimally required sample sizes that are needed to discriminate FGR from the CTRL and/or SGA individuals on the basis of the obtained data with the help of the G*Power statistical software (version 3.1.9.2, University of Düsseldorf) [31]. A type I error (α) of 0.05 and a type II error (β) of 0.20 were chosen in a comparison of two means. The minimally required power (1-β error probability) was 0.80.

## 3. Results

### 3.1. Patient Cohorts and MALDI Mass Spectrometric Profiling

The University Hospital Aachen is a tertiary care center with a high percentage of high-risk pregnancies. Between August 2006 and November 2011, women with singleton pregnancies attending the Department of Obstetrics and Gynecology for any reason between 24 and 40 weeks of gestation were asked to participate in a prospective observational study for biomarker development for the detection of FGR and preeclampsia. No specific situation was considered, however most of the patients were admitted to the outpatient clinic for routine checks or planning of birth or because of suspected preterm birth, suspected FGR, or suspected preeclampsia. Of the approximately 5000 women who delivered at the University Hospital Aachen during the recruitment period, ca. 10% (531 individuals) agreed to participate in the study. Of those, 167 patients fell into the group of suspected pregnancies with fetal weight below the 10th percentile. In 95 patients, gestational age was 34 weeks and below. Finally, peripheral venous blood samples from 75 Caucasian singleton pregnancies were chosen from the biobank to be subjected to blood serum proteome analysis by affinity-mass spectrometry.

Women with normal pregnancies (cohort CTRL I) delivered healthy infants with adequate for gestational age neonatal weight, i.e., within the 10th and 90th percentile at the expected gestational age of ca. 40 weeks, and hence, represented the general population. The mean time difference between blood sampling and delivery was 71 days. Eleven of the 15 women from cohort CTRL II delivered preterm for various reasons (premature rupture of the membrane, spontaneous onset of labor, vaginal bleeding). The mean time difference between blood sampling and delivery was 3 days. From the FGR I cohort all 15 women and from the FGR II cohort, 13 out of 15 women needed mandatory preterm delivery for non-reassuring fetal well-being. The mean time difference between blood sampling and delivery was 5 and 6 days, respectively. The mean days SGA babies (cohort SGA I) were born after blood sampling was 50 days. Maternal age, BMI, and blood pressure did not differ significantly between groups, as indicated by the overlap of 95% CI. Most of the women in all groups were primiparous. Women within the newly added FGR I cohort were more likely to smoke. As per definition, birth-weight percentiles differed significantly between the FGR/SGA and CTRL groups, as indicated by the non-overlap of the 95% confidence interval (Table 1 and Supplemental Table S1).

**Figure 1:**
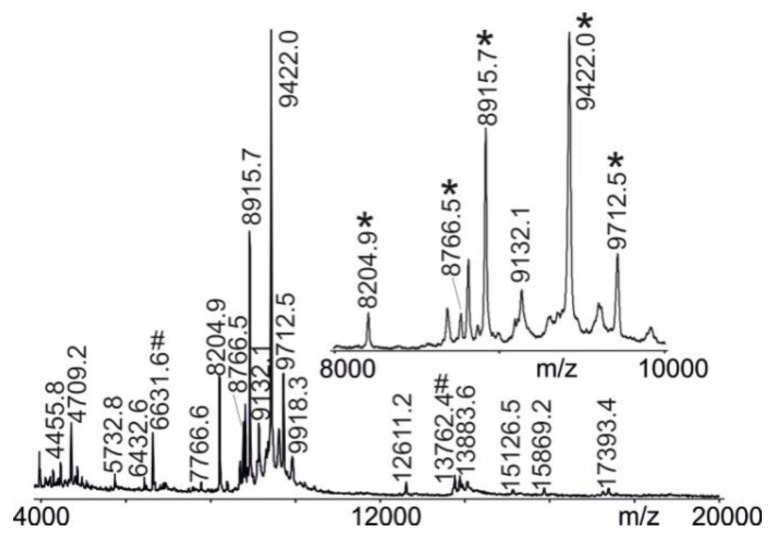
Affinity—MALDI-ToF mass spectrum of intact protein ions. Proteins are from maternal peripheral venous blood serum upon work-up with ClinProt^®^ beads. Ion signals that are selected for multi-parametric analysis are labeled with “*” (see insert). Ion signals that are used for internal re-calibration are marked with “#”. Ferulic acid is used as the matrix.

Following our standardized protocol for producing protein solutions out of blood samples, on average, about 60 protein ion signals were reproducibly recorded in each mass spectrum within a mass range of m/z 4000 to m/z 20,000 (Figure 1). The most prominent ion signals were observed in the mass range between m/z 8000 and m/z 10,000 (Figure 1, insert), correlating to singly charged (protonated) ion signals of small proteins. The ion signals of which their areas were used for bio-statistical analysis were, as in our previous study [7,11], from apolipoprotein CII (m/z 8205), pro-apolipoprotein CII (m/z 8916), apolipoprotein CIII_0_ (m/z 8766), apolipoprotein CIII_1_ (m/z 9422), and apolipoprotein CIII_2_ (m/z 9713). 

The MALDI-ToF mass spectra from proteins of FGR, CTRL, and SGA serum samples displayed high similarities to each other, indicating that relative quantitative differential analysis of ion signal intensities, and of quotients thereof, was feasible. Exceptions were mass spectra from patients 115 and 315, respectively. These were excluded from further analysis to leave a PPS with 206 mass spectra from 73 individuals (Scheme 1).

### 3.2. Determination of “Best Cut-Off” Values for Quotients A, B, and C to Separate FGR from CTRL

Areas of the five selected ion signals were determined and brought into context with each other following the previously introduced rules, thereby generating quotients A, B, and C. Then, “best cut-off” values for quotients A, B, and C were to be applied in our assay to assign a given mass spectrum (patient sample) to one of the clinical groups, i.e., FGR, SGA, or CTRL. However, because the serum protein composition of the CTRL I cohort was not yet investigated, we decided not to use the “best cut-off” values from our previous study [7], although quotients A, B, and C were determined in the same way as before, using the areas of the same ion signals as was done previously. Instead, we generated a training set “O”, which contained 14 mass spectra for CTRL I (series MS1) and 14 mass spectra for FGR I (series MS1) (Supplemental Table S2 and Supplemental Scheme S1). Jmax values indicated the “best cut-off” values of 4.2, 5.0, and 4.0 for quotients A, B, and C, respectively (Supplemental Tables S3–S5). Next, the cumulative score for each mass spectrum was calculated according to previously established rules: a score of “1” was assigned to this respective spectrum (sample) when the quotient value of this specific spectrum (sample) was higher than the respective “best cut-off” value. In the contrary case, the score for this spectrum (sample) was set to “0”. These assessments were again independently carried out for each of the three ion signal ratios and for each spectrum. In sum, each sample reached a cumulative score between “0” and “3”, as was the case in our previous study [7].

**Figure 2:**
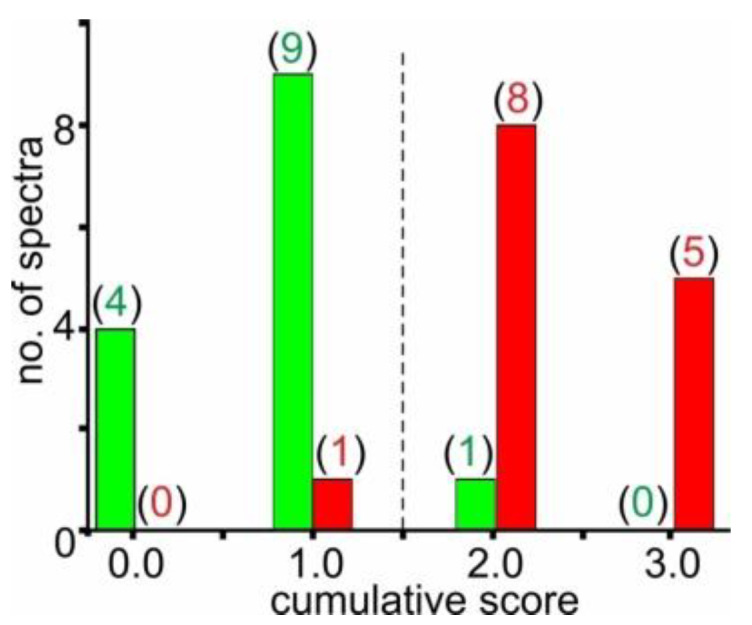
Distribution pattern of cumulative scores with training set “O”. Cumulative score values range between “0.0” and “3.0”. Spectra of training set “O” (first measurement series, MS1, of CTRL I and FGR I serum samples; *n* = 28) are represented. Green bars represent spectra of CTRL I (patients 101–114) and red bars represent spectra of FGR I (patients 301–314, see Supplemental Table S2). Numbers in parentheses indicate numbers of spectra with the respective cumulative score. The vertical dashed line marks the cumulative score cut-off value which sorts the spectra, which is the respective samples, into either the FGR group (right) or the CTRL group (left).

Of note, the cumulative score discriminator was kept at 1.0, meaning a cumulative score below or equal to 1.0 assigned a given mass spectrum to the CTRL group and a cumulative score above 1.0 to the FGR group. For training set “O”, the distribution of cumulative scores (Figure 2) revealed that one of the 14 FGR spectra were wrongly assigned to the CTRL group and one of the 14 CTRL spectra were wrongly placed in the FGR group.

Accordingly, excellent bio-statistical results were obtained, i.e., sensitivity was 0.93 and specificity was 0.93. Hence, an area under curve (AUC) in the receiver-operator characteristics (ROC) analysis of 0.95 was reached (Table 2). The cumulative score distribution was applied for power analysis investigations, which showed that the required minimal sample size was 3 FGR and 3 CTRL mass spectra to reach a statistically meaningful separation of the two groups (Supplemental Table S6).

### 3.3. Combination of “Best Cut-Off” Values and Weighting of Cumulative Scores for Separating FGR from CTRL

**Figure 3:**
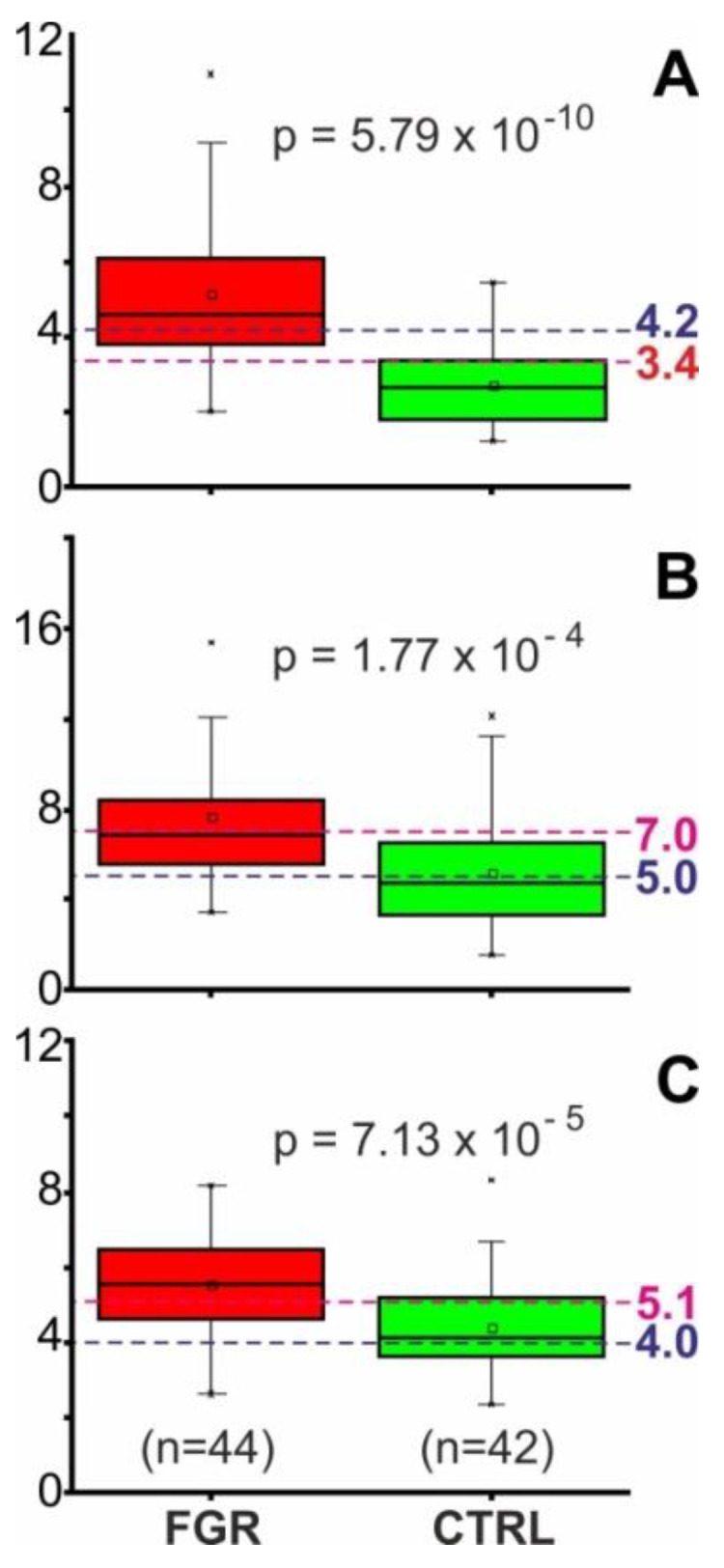
Distribution of quotient values for selected ion intensity differences of the “development” test set. Quotient values distributions are shown as box and whisker plots (second measurement series, MS2, of CTRL I and FGR I plus third and fourth measurement series, MS3 and MS4, of CTRL II and FGR II serum samples, *n* = 86). (**A**) Signal area of the ion at m/z 8916 over the signal area of the ion at m/z 8205. (**B**) Signal area of the ion at m/z 8766 over the sum of the signal areas of ions at m/z 9422 plus m/z 9713. (**C**) Signal area of the ion at m/z 8916 over the sum of signal areas of ions at m/z 8766 plus m/z 9422 plus m/z 9713. The boxes represent the 25th–75th percentiles. The horizontal lines within the boxes represent the medians; the small squares indicate the means. The whiskers specify the 5th and 95th percentiles and the crosses indicate the 1st and 99th percentiles. Dashed lines mark selected cut-off values. Cut-off values for the quotients are (**A**) 3.4 and 4.2, (**B**) 5.0 and 7.0, (**C**) 4.0 and 5.1. FGR, fetal growth restriction; CTRL, control individuals.

Since the “best cut-off” values of 4.2, 5.0, and 4.0 for quotients A, B, and C, respectively, which were obtained in this study with training set “O”, were somewhat different from those “best cut-off” values of 3.4, 7.0, and 5.1 for quotients A, B, and C, respectively, from our previous study (training set “W”) [7], we decided to apply both sets of “best cut-off” values in combination when analyzing the test sets. The “development” test set (Supplemental Scheme S1) contained mass spectra from CTRL I (series MS2) and from FGR I (series MS2) as well as spectra from CTRL II (series MS3 and MS4) and from FGR II (series MS3 and MS4), summing up to 42 mass spectra for CTRL (28 women) and 44 mass spectra for FGR (29 patients). The quotient value distributions were found to be distinctive, such that the values for quotients A, B, and C were generally higher in the FGR group as compared to those of the CTRL group (Figure 3).

Since the application of two “best cut-off” values per quotient generated three value regimes (below—in between—above), the score of a mass spectrum was assigned “0.0” when falling into the regime “below”, “0.5” when “in between”, and “1.0” when “above”. Accordingly, the cumulative score values of each spectrum, summed up from all three ion signal ratios, ranged between “0.0” and “3.0” with steps of 0.5. Keeping the cumulative score discriminator at 1.0, as was done above and in our previous studies, decided whether a given mass spectrum was assigned to the CTRL group or to the FGR group. 

**Figure 4:**
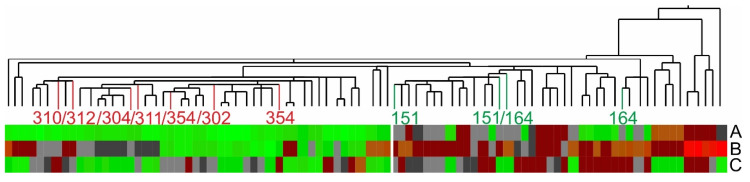
Hierarchical clustering analysis with quotient values A, B, and C of protein ion signal areas of the “development” test set. This set contains the second measurement series, MS2, of CTRL I and FGR I plus the third and fourth measurement series, MS3 and MS4, of CTRL II and FGR II serum samples (*n* = 86). Quotients A to C are arranged from top to bottom. Color code for the quotient values; red: high values; brown: medium high; grey: medium low; green: low. Patient numbering is as in Supplemental Tables S1 and S2.

Using quotient values A, B, and C from the “development” test set separated the FGR group (*n* = 44) from the CTRL group (*n* = 42) quite satisfactorily by hierarchical clustering (Figure 4), confirming that the ion signal abundances of five proteins were carrying the requested information for differentiating FGR from CTRL with good confidence. Duplicate measurements from FGR samples are clearly sorted to the FGR group (right) and control samples to the CTRL group (left), except for FGR samples 302, 304, 310, 312, and 364 (one measurement each), which are allocated to the CTRL group, and CTRL samples 151 (both measurements) and 164 (one measurement) are grouped to the FGR group.

Bio-statistic evaluation of the “development” test set performance revealed a false positive rate of 0.21 and a false negative rate of 0.10. Hence, an area under curve (AUC) in the receiver-operator characteristics (ROC) analysis of 0.88 was reached (Table 2). The cumulative score distribution was applied for power analysis investigations, which showed that the required minimal sample size was 9 FGR and 9 CTRL mass spectra to reach a statistically meaningful separation of the two groups (Supplemental Table S6).

### 3.4. Application of “Weighted Cumulative Scores” for Separating FGR from CTRL and from SGA

Encouraged by the separation power with which pregnant women whose fetuses suffered from FGR could be distinguished from CTRL individuals whose pregnancies were unaffected, solely based on ion signal abundances of serum proteins as recorded in MALDI mass spectra, we generated a “validation” test set (Scheme 1 and Supplemental Scheme S1) which contained 118 mass spectra from three different patient/donor groups (73 individuals). Because of indeterminate results, 4 mass spectra (patients 155 and 213) were excluded, leaving 114 mass spectra (71 individuals) for the biostatistics analysis. The FGR group contained 44 mass spectra (29 patients), 14 from cohort FGR I (series MS2) and 30 from cohort FGR II (series MS3 and MS4). The CTRL group contained 42 mass spectra (28 individuals), 14 from cohort CTRL I (series MS2) and 28 from cohort CTRL II (series MS3 and MS4). The SGA group contained 28 mass spectra (series MS 1 and MS2) from cohort SGA I (14 patients). The analysis procedure followed what was described above for the “development” test set and started with determining the quotient values A, B, and C, respectively.

**Figure 5:**
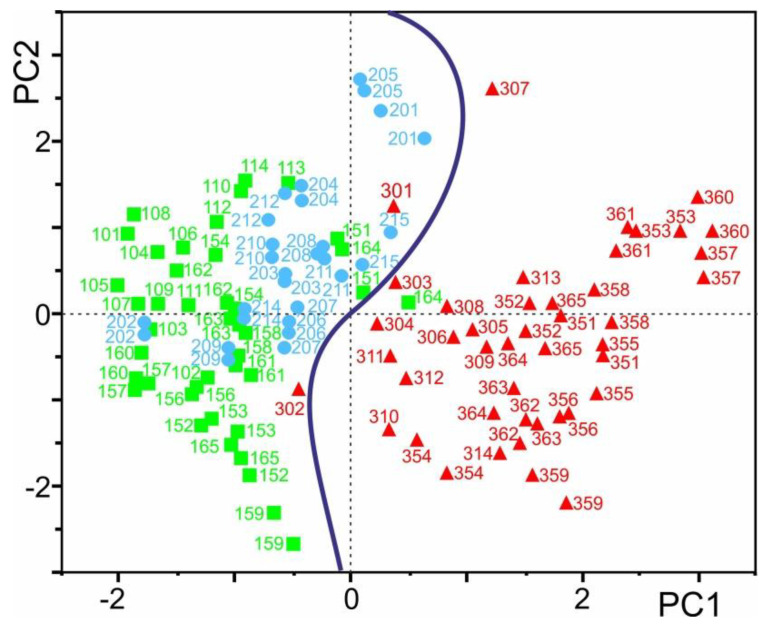
Principle component analysis with quotient values A, B, and C of protein ion signal areas of the “validation” test set. This set contains measurement series MS2 of cohorts CTRL I and FGR I plus measurement series MS3 and MS4 of cohorts CTRL II and FGR II, in addition to measurement series MS1 and MS2 of cohort SGA I serum samples (*n* = 114) which represent 71 individuals. Locations of mass spectra derived positioning from FGR samples are shown as red triangles, locations of CTRL samples as green squares, and SGA patients as blue circles (filled symbols). The curved line indicates the decision boundary obtained from the support vector machine (SVM) algorithm classifier.

Subjecting the quotient values (in total, 342 values) to Principal Component Analysis (PCA) afforded two well separated clusters. The first and second centered PCs of the quotient data yielded 48.6% (PC1) and 27.5% (PC2) of the total variances, respectively. The decision boundary, which was obtained from the SVM classifier, separated both clusters with the exceptions of FGR samples 301 and 302, which were placed on the CTRL side. Likewise, CTRL sample 164 was placed on the FGR side. It should be mentioned that SGA individuals clustered with the CTRL donors and, hence, were separated from FGR patients with good confidence (Figure 5).

In agreement with the obtained PCA results, good separation was achieved with the cumulative score discriminator of 1.0 as well. From the 71 individuals (114 mass spectra) of the “validation” test set, 59 were assigned correctly (26 TP and 33 TN), i.e., their grouping stood in agreement with the clinical assignment which served as the “gold standard”. The positive predictive value was 0.74 and the negative predictive value 0.92, hence an area under curve (AUC) in the receiver-operator characteristics (ROC) analysis of 0.88 was reached (Table 2). The cumulative score distribution was applied for power analysis investigations, which showed that the required minimal sample size was 5 FGR and 5 CTRL/SGA mass spectra to reach a statistically meaningful separation of the two groups (Supplemental Table S6).

## 4. Discussion

The aim of the present study was to establish a blood-based biomarker test to detect FGR and to distinguish from constitutional SGA that is robust, simple, and easily available and may be added to current clinical practice to improve detection rates. The study took advantage of an existing biobank in which patients with suspected SGA/FGR and CTRL were included and well characterized by antenatal ultrasound inspection. Maternal serum proteins were analyzed by affinity mass spectrometry at the time point of admission to hospital. Our test discriminated between FGR and SGA as well as pregnancies unaffected by FGR, i.e., CTRL, with good confidence. The use of two cut-off values for each quotient of ion signal areas opened three value regimes: below—in between—above cut-offs. Despite differing from rather routinely used two value regimes (below—above cut-offs) for separation of samples/individuals, separation of FGR from CTRL/SGA was successfully employed. Examples of other clinical studies in which it was found that a three value regime was suitable for fulfilling the task to separate two conditions are “early risk prognosis of free-flap transplant failure”, “MGMT promoter methylation for selecting glioblastoma patients into trials omitting Temozolomide”, and “diagnostic criteria for high-dimensional metabolic data in newborn screening for medium-chain acyl-CoA dehydrogenase deficiency” [22,32,33], but also with sFlt-1/PlGF ratio (<38 and >85) [34,35] and PlGF measurements alone to predict still-birth FGR (<12 and >100) [36].

Our study is limited by the small sample size. Moreover, a selection bias of samples taken from the biobank cannot be excluded. Selection of suitable control groups for “case-control” clinical studies is very important and sometimes critical for the distinguishing ability of the assay [37,38]. Since it is known that maternal blood protein compositions change in complexity and abundance with advancing gestational age [39,40,41], such reasoning becomes critical with any blood protein-based assay that shall find application in pregnant women. Adaptation of cut-off values depending on gestational age has already found application in point-of-care diagnostics, which is based on abundance ratios of molecular markers found in maternal blood [42].

Strengths of our study are the well characterized patient cohorts and the application of multiparametric mass spectrometry measurements. A combination of marker proteins, i.e., angionenic factors and acute-phase proteins in serum samples, was found to yield in good discrimination of HELLP and preeclampsia from control [43]. The need for accumulating markers for screening purposes sooner or later may request to move away from immunoanalytical assays, such as ELISAs, and orient towards screening systems with inherent multiplexing capabilities. Affinity-mass spectrometry, as performed here, enables parallel analysis of dozens of proteins and accurate determination of relative protein abundances as well as ratios of, for example, differently modified protein species, which allow to differentiate varying glycosylation and other post-translational modifications simply by mass, but which may be difficult to detect and/or to differentiate by conventional antibody assays [18]. 

Current state of the art FGR detection is based on ultrasound assessment. However, even in high-income countries, like Germany, in which antenatal ultrasound is offered most frequently and usually routinely more than 3 times during pregnancy, FGR detection rate ranges between about 20–50% [8,9]. A high percentage of undetected cases lead to sub-standard care, stillbirth, and increased risk of perinatal mortality and morbidity. Moreover, diagnosis is often delayed because estimating fetal growth velocity needs to be performed at two different time points during pregnancy, which take place at least 14 days apart [44]. Diagnosis requires both availability of ultrasound equipment and ultrasound trained specialists [1,2,45]. Although stillbirth remains an important clinical issue for high-income countries, the majority of cases occur in low- and middle-income countries where ultrasound inspection is hardly available. Currently used clinical tests in these countries, such as measurement of symphysis-fundal height, may have even lower sensitivity and specificity for the identification of SGA infants—the primary step in diagnosing FGR—than ultrasound assessments [46]. Since ultrasound-based investigations obviously lack power, effort has been given to improve diagnosis, for example, by adding single marker proteins, such as PlGF-measurements, to standard care. The PELICAN trial aimed at evaluating the diagnostic accuracy of PlGF in women with suspected preeclampsia. They also challenged PlGF measurements for detecting SGA with birth weight below the 1st percentile. Using a PlGF cut-off below the 5th percentile for gestational age sensitivity ranged between 0.91 to 0.93, and specificity between 0.51 and 0.53 (before or after 35 weeks of gestation, respectively), resulting in a high degree of false positive classified patients [47]. Similarly, Benton et al. challenged PlGF measurements in 219 patients with antenatally suspected FGR defined as a fetal abdominal circumference (AC) <10th percentile for gestational age on ultrasound. In their cohorts from Canada, New Zealand, and the United Kingdom, which also included samples of the PELICAN trial, they found a sensitivity of 0.98 and a specificity of 0.75 for the antenatal identification of FGR using the 5th percentile of PlGF as a cut-off. They also highlighted that PlGF levels correlate with the degree of placental pathology in these patients [48]. Likewise, the addition of sFlt-1 to PlGF measurement has been suggested for the detection of high-risk pregnancies. An sFlt-1/PlGF ratio below 38 has been proven to be capable for ruling out pathologic pregnancies like preeclampsia [34,35]. In FGR pregnancies, sFlt-1/PlGF ratios are increased [34] and in a recent observational trial, Quezada et al. reported sFlt-1/PlGF ratios above 85 in 75% of patients with diagnosed FGR [49]. In the study of Visan et al., the combination of sFLT1/PIGF ratio at a cut-off of 38 to ultrasound-based estimation of fetal weight <10th percentile led to an increase in sensitivity for the detection of FGR from 44.4% to 84.2%, with a change in specificity from 89% to 84.3%, with a false-positive rate of 10% [10].

Similar to others, with our assay, we found a relatively higher rate (9 out of 71) of false positive classified patients with respect to ultrasound data (“gold standard”). A high false positive rate causes unnecessary anxiety of pregnant women and increases rates of intervention. Yet, time of uncertainty can be considered rather short (approximately 3–4 days) as in true positive cases, delivery is expected to take place rather soon after testing. Hence, if pregnancy continues for more than 5 days after testing, a repeated test may be scheduled to confirm or falsify the primary test result. 

Obviously, earlier confirmation of placental dysfunction in suspected fetal growth restriction has the potential to improve risk stratification and earlier access to targeted surveillance. More studies combining ultrasound and blood-borne biomarkers are needed to determine whether this approach improves diagnostic accuracy over the use of ultrasound estimation of fetal size or biochemical markers of placental dysfunction alone [50]. Our multiparametric affinity-mass spectrometry test may aid in screening and in decision-making processes, e.g., to refer patients to ultrasound specialists, especially in rural areas with sub-standard care. 

## 5. Conclusions

In conclusion, we have developed an affinity-mass spectrometry-based biomarker test for the detection of FGR in pregnant women, which was challenged against the actual gold standard of antenatal ultrasound-based diagnosis. Our approach allows for a multi-marker-based screening in a single blood test, which has been proven to be robust and easy to perform and allows FGR risk assessment with high confidence. The combination of this blood test with clinical examination offers a promising means for better antenatal care, which shall be further evaluated in follow-up multi-centric studies.

## Figures and Tables

**Table 1:** Summarized or averaged demographic data as well as clinical and laboratory parameters for all patients and control individuals.

Parameter	CTRL I (101–115) ^i^					CTRL II (151–165) ^j^					FGR I (301–315) ^i^					FGR II (351–365) ^j^					SGA I (201–215) ^i^				
	*n*	Mean	95% CI	Min.	Max.	*n*	Mean	95% CI	Min.	Max.	*n*	Mean	95% CI	Min.	Max.	*n*	Mean	95% CI	Min.	Max.	*n*	Mean	95% CI	Min.	Max.
**Mat. Age (years) ^a^**	15	29.4	26.5–32.3	21.5	39.2	15	30.5	27.7–33.3	24.2	39.0	15	28.1	25.1-31.1	22.0	41.8	15	30.4	26.8-34.0	19.1	41.5	15	28.9	24.9–32.8	17.6	40.2
**Mat. BMI, (kg/m^2^) ^b^**	15	22.8	21.7–23.9	19.5	26.9	15	23.3	20.4–26.2	17.6	35.7	15	22.2	20.6–23.9	18.0	28.9	15	24.2	22.4–26.1	19.9	31.9	15	22.4	20.6–24.3	16.2	28.1
**Primiparity, (%)**	15	100.0	n.a.	n.a.	n.a.	15	80.0	n.a.	n.a.	n.a.	15	93.3	n.a.	n.a.	n.a.	15	86.7	n.a.	n.a.	n.a.	15	86.7	n.a.	n.a.	n.a.
**Smoking Status, (%)**	15	20.0	n.a.	n.a.	n.a.	15	20.0	n.a.	n.a.	n.a.	15	46.7	n.a.	n.a.	n.a.	15	26.7	n.a.	n.a.	n.a.	15	26.7	n.a.	n.a.	n.a.
**Systolic bp, (mmHg) ^c^**	15	115.6	109.2–122.0	99	135	15	116.2	121.1–120.3	106	129	15	120.4	113.6–127.2	96	139	15	130.9	118.6–143.1	83	162	15	117.5	108.9–126.2	84	146
**Diastolic bp, (mmHg) ^c^**	15	68.9	63.1–74.6	54	87	15	64.9	60.5–69.4	53	81	15	69.1	63.6–74.5	52	82	15	77.3	68.2–86.3	52	101	15	66.3	60.2–72.3	45	84
**ga at sample, (weeks) ^d^**	15	29.4	28.3–30.5	25.7	32.6	15	32.4	29.8–35.0	25	39.3	15	29.6	28.3–30.9	25.4	33.9	15	32.3	29.7–34.9	24	40.4	15	31.0	29.3–32.7	22.9	34.6
**ga at delivery, (weeks) ^d^**	15	39.6	38.9–40.3	37.4	41.6	15	32.8	30.2–35.3	25	39.3	15	30.5	29.2–31.8	25.9	34.1	15	33.0	30.5–35.5	26.7	40.4	15	38.1	37.4–38.8	36.1	40.7
**Δt (days) ^e^**	15	71.3	62.7–79.8	50	105	15	2.7	0.01–5.3	0	18	15	6.3	2.1–10.6	0	31	15	4.7	0.95–8.4	0	19	15	49.9	37.1–62.8	22	105
**C-section, (%) ^f^**	15	20.0	n.a.	n.a.	n.a.	15	86.7	n.a.	n.a.	n.a.	15	100.0	n.a.	n.a.	n.a.	15	100.0	n.a.	n.a.	n.a.	15	66.7	n.a.	n.a.	n.a.
**Fetal Birth Weight, (g)**	15	3399	3232–3565	2960	3765	15	2129	1584–2673	740	3895	15	1015	845–1185	495	1525	15	1383	1005–1761	490	2665	15	2465	2299–2630	1950	2780
**fbw Percentile ^g^**	15	47.1	37.5–56.8	14	70	15	50.7	40.8–60.5	25	85	15	5.2	3.8–6.6	2	9	15	4.0	2.5–5.5	1	9	15	4.5	3.2–5.7	1	9
**Female, (%) ^h^**	15	60.0	n.a.	n.a.	n.a.	15	53.3	n.a.	n.a.	n.a.	15	40.0	n.a.	n.a.	n.a.	15	53.3	n.a.	n.a.	n.a.	15	46.7	n.a.	n.a.	n.a.

(a) mat: maternal age in years determined at the beginning of hospitalization (time point of blood sampling); n.a.: not applicable; (b) pre pregnancy BMI; (c) blood pressure; (d) gestational age; (e) time interval between sample collection and delivery; (f) c: caesarea, mode of delivery; (g) fbw: fetal birth weight; (h) fetal gender; (i) this study; (j) from [7].

**Table 2:** Summary of biostatistic evaluation of individual patients ^a^.

No	Type of Data Set/Cohort	TP	FP	TN	FN	Sens	Spec	FPR	FNR	PPV	NPV	ROC AUC
1	**training “O”/CTRL I vs. FGR I** (CTRL I, *n* = 14; FGR I, *n* = 14) ^b^	13	1	13	1	0.93	0.93	0.07	0.07	0.93	0.93	0.95
2	**“development” test/CTRL I + II vs. FGR I + II** (CTRL, I + II, *n* = 28; FGR I + II, *n* = 29) ^c^	26	6	22	3	0.90	0.79	0.21	0.10	0.81	0.88	0.88
3	**“valid” test/CTRL I + II and SGA I vs. FGR I + II** (CTRL I + II, *n* = 28; SGA I, *n* = 14; FGR I + II, *n* = 29) ^c^	26	9	33	3	0.90	0.79	0.21	0.10	0.74	0.92	0.88

(a) cumulative score separator: > 1 = FGR; ≤ 1 = CTRL; (b) no. of mass spectra; Jmax determined cut-off values; (c) no. of patients; combined cut-off values; TP: true positive; FP: false positive; TN: true negative; FN: false negative; sens: sensitivity; spec: specificity; FPR: false positive rate; FNR: false negative rate; PPV: positive predictive value; NPV: negative predictive value; ROC: receiver operating characteristic; AUC: area under the curve.

## Supplementary Materials

The following are available online at https://www.mdpi.com/2077-0383/9/5/1374/s1, Scheme S1: Disease groups and patient cohorts; Table S1: Demographic Data, Clinical and Laboratory Parameters for all Patients and Control Individuals; Table S2: Areas of ion signals selected for scoring; Table S3: Determination of Youden Index (Jmax) for Quotient A; Table S4: Determination of Youden Index (Jmax) for Quotient B; Table S5: Determination of Youden Index (Jmax) for Quotient C; Table S6: Power analysis of mass spectrometric ion signal-derived proteome profile series.

## Abbreviations

ACOG: American College of Obstetricians and Gynecologists
MALDI ToF MS: Matrix-Assisted Laser Desorption/Ionization Time of Flight Mass Spectrometry
m/z: mass to charge ratio
