# Supplementary material for: Precision Diagnostics by Affinity-Mass Spectrometry: A Novel Approach for Fetal Growth Restriction Screening during Pregnancy
Source: J Clin Med. 2020 May 7;9(5):1374. doi: 10.3390/jcm9051374 (PMC7290972; doi:10.3390/jcm9051374)
Supplement: Supplementary file 1 [file jcm-09-01374-s001.pdf]

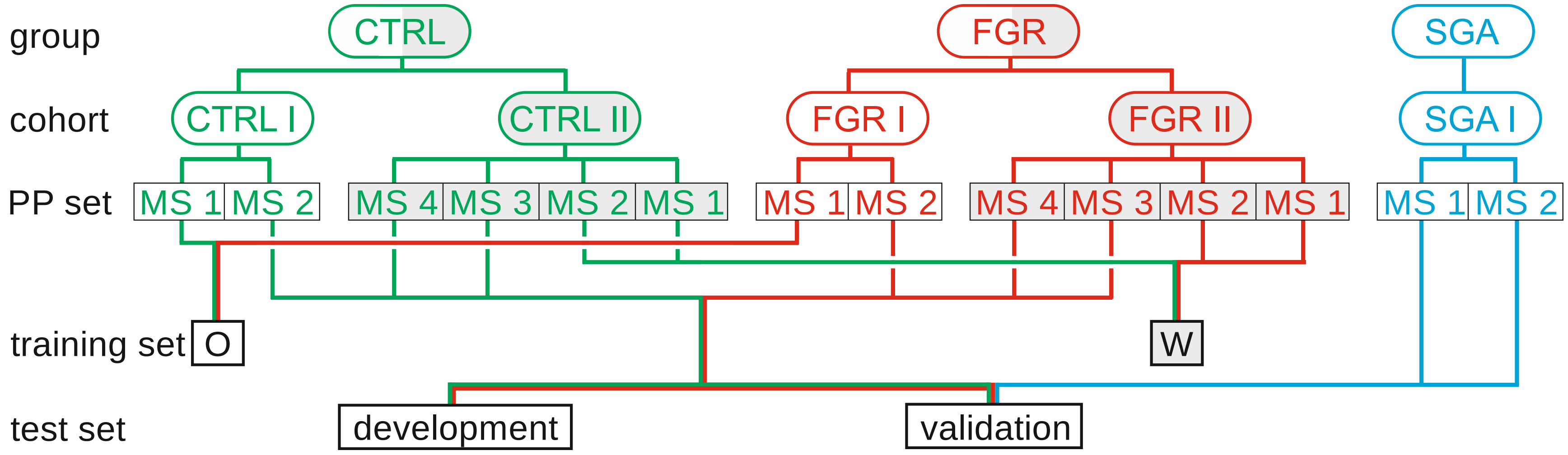

Supplemental Scheme 1: The three disease / control groups are represented by five patient cohorts. Individuals for cohorts CTRL I, FGR I, and SGA I have been recruited for this study, whereas cohorts CTRL II and FGR II consisted of individuals which were included in a previous study as well . The training sets “O” (this study) and “W” (from) were both used for determining cut-off values and the “development” test set for estimating assay performance. With the “validation” test set was investigated the assay´s capability to separate SGA from FGR in the presence of CTRL samples.

Supplemental Table 1: Demographic Data, Clinical and Laboratory Parameters for all Patients and Control Individuals

	mothers												infants		
	patient / individual	age [a)]	BMI [kg/m^2]	parity [a)]	median blood pressure [a.b)] [mmHg]	proteinuria [mg/l] [a.c.d)]	smoking status [e)]	co-morb. [f)]	gestational age at collection of blood [w+d] [g)]	gestational age at delivery [w+d] [g)]	Δd[h)]	mode of delivery [i)]	birth weight [g]	birth weight percentile	sex
CTRL I	101	35.0	20.4	0	108 / 60	0	0	/	29 + 4	39 + 2	68	s.c.	3210	40	f
	102	23.6	22.0	0	102 / 54	0	0	/	30 + 6	41 + 2	73	vaginal	3765	50	m
	103	28.9	20.3	0	120 / 60	0	0	/	32 + 4	41 + 4	63	vaginal	3580	50	f
	104	21.5	23.9	1	115 / 60	0	20	/	30 + 4	37 + 6	51	s.c.	3200	43	m
	105	31.7	23.9	1	110 / 70	0	0	/	28 + 5	40 + 0	79	vaginal	3690	60	m
	106	28.9	22.2	0	122 / 68	0	0	/	31 + 6	39 + 0	50	vaginal	3420	60	f
	107	39.2	21.1	1	127 / 61	0	0	/	31 + 3	39 + 0	53	vaginal	3440	60	f
	108	32.0	24.1	0	130 / 80	0	0	/	30 + 4	39 + 3	62	vaginal	3050	20	f
	109	25.1	26.9	0	99 / 87	0	0	/	27 + 6	37 + 3	67	vaginal	2960	30	m
	110	29.3	23.5	0	135 / 70	0	0	/	28 + 4	39 + 1	74	s.c.	3450	50	m
	111	31.2	23.2	1	116 / 81	0	0	/	28 + 0	41 + 0	91	vaginal	3750	70	f
	112	26.6	19.5	0	100 / 80	0	0	/	30 + 2	39 + 4	65	vaginal	3660	60	m
	113	24.5	22.4	1	115 / 59	0	2	/	26 + 2	38 + 2	84	vaginal	2970	30	f
	114	38.2	25.5	1	130 / 80	0	0	/	25 + 5	40 + 5	105	vaginal	3765	70	f
	115 [j)]	25.7	23.1	1	105 / 63	0	25	/	28 + 4	40 + 4	84	vaginal	3070	14	f
CTRL II	151	24.9	20.3	2	109 / 53	0[f]	20	/	27+0	27+4	4	s.c.	1355	85	m
	152	37.9	23.1	2	108 / 65	0[f]	0	/	27+1	27+4	3	s.c.	1095	40	m
	153	30.5	22.1	0	114 / 61	0[f]	0	/	25+0	25+0	0	vaginal	740	30	m
	154	30.3	20.8	1	124 / 69	0[f]	0	/	31+2	33+6	18	s.c.	1960	25	f
	155	29.9	21.4	0	114 / 71	150	0	/	32+0	32+1	1	s.c.	1920	50	m
	156	24.2	35.7	0	129 /73	0[f]	10	/	31+6	31+6	0	vaginal	1750	50	f
	157	32.1	22.4	0	126 / 63	0[f]	0	/	37+4	37+4	0	s.c.	3130	50	f
	158	31.8	25.8	1	121 / 75	0[f]	0	/	37+5	37+5	0	s.c.	3465	70	m
	159	35.7	21.8	1	122 / 58	0[f]	0	/	33+6	34+6	7	s.c.	2420	40	f
	160	37.1	19.1	1	120 / 60	0[f]	0	/	38+6	39+0	1	s.c.	3150	30	f
	161	40.0	20.8	1	110 / 60	0[f]	0	/	36+0	36+0	0	s.c.	2920	60	m
	162	32.7	25.1	2	110 / 70	0[f]	0	/	32+3	32+3	0	s.c.	1850	50	f
	163	29.6	24.1	1	106 / 61	0[f]	0	/	27+5	28+4	6	s.c.	1250	60	f
	164	24.6	17.6	1	110 / 54	0[f]	5	/	28+0	28+0	0	s.c.	1030	40	f
	165	24.7	35.4	1	120 / 81	0[f]	0	/	39+2	39+2	0	s.c.	3895	80	m

Supplemental Table 1 (continued)

	mothers												infants		
group	patient / individual	age [a)]	BMI [kg/m^2]	parity [a)]	median blood pressure [a.b)] [mmHg]	proteinuria [mg/l] [a.c.d)]	smoking status [e)]	co-morb. [f)]	gestational age at collection of blood [w+d] [g)]	gestational age at delivery [w+d] [g)]	Δd [h)]	mode of delivery [i)]	birth weight [g]	birth weight percentile	sex
IUGR I	301	41.8	23.7	3	134 / 78	0	0	/	33 + 6	34 + 1	2	s.c.	1525	3	m
	302	34.9	19.9	0	130 / 82	0	0	/	29 + 3	30 + 0	4	s.c.	755	2	m
	303	31.9	22.5	0	129 / 76	90	0	/	31 + 0	32 + 1	8	s.c.	1255	9	f
	304	24.5	19.7	0	122 / 53	0	40	/	26 + 1	26 + 4	3	s.c.	590	3	m
	305	28.8	26.0	1	120 / 80	0	15	/	30 + 5	31 + 5	7	s.c.	1190	6	m
	306	27.5	22.1	0	130 / 68	0	0	/	32 + 6	33 + 2	3	s.c.	1405	6	f
	307	30.7	24.0	0	120 / 75	0	10	/	28 + 1	28 + 5	4	s.c.	730	4	f
	308	29.4	20.1	0	103 / 59	0	0	/	29 + 6	31 + 4	12	s.c.	1240	9	m
	309	23.6	23.7	0	125 / 71	101	10	/	30 + 5	30 + 5	0	s.c.	940	4	f
	310	27.8	18.0	1	111 / 64	0	3	/	27 + 1	28 + 6	12	s.c.	900	8	m
	311	22.0	19.4	0	110 / 60	0	0	/	25 + 3	25 + 6	3	s.c.	495	3	m
	312	28.7	19.2	0	128 / 76	0	0	/	28 + 4	33 + 0	31	s.c.	1245	4	f
	313	23.4	23.5	0	96 / 52	0	5	/	29 + 3	29 + 5	2	s.c.	855	4	m
	314	23.1	29.0	0	109 / 77	0	5	/	29 + 4	29 + 6	2	s.c.	860	4	f
	315 [j)]	23.4	22.7	1	139 / 65	0	0	/	31 + 2	31 + 4	2	s.c.	1240	9	m
IUGR II	351	31.9	22.5	0	129 / 76	90	0	/	31+0	32+1	8	s.c.	1255	9	f
	352	23.6	23.7	0	125 / 71	101	10	/	30+5	30+5	0	s.c.	940	4	f
	353	33.1	31.9	0	155 / 100	2324	10	PE	24+0	26+5	19	s.c.	650	4	m
	354	25.6	26.2	0	83 / 52	0[f]	0	/	37+3	37+5	2	s.c.	2550	5	m
	355	39.6	27.1	0	148 / 92	300	0	PE	28+2	28+2	0	s.c.	850	8	m
	356	34.3	19.9	0	136 / 71	0[f]	0	/	32+0	32+1	1	s.c.	1310	8	m
	357	23.8	21.5	0	162 / 100	500	0	PE	35+5	35+5	0	s.c.	1330	1	f
	358	27.5	22.1	0	130 / 68	150	0	/	32+6	33+2	3	s.c.	1405	6	f
	359	19.1	23.5	0	116 / 64	0[f]	10	/	35+5	36+1	3	s.c.	1870	2	m
	360	27.8	28.3	3	112 / 55	0[f]	0	/	27+0	27+0	0	s.c.	580	3	f
	361	31.7	20.2	1	100 / 60	0[f]	0	/	38+6	39+0	1	s.c.	1970	1	f
	362	34.0	23.8	2	140 / 90	1725	0	PE	26+4	26+6	2	s.c.	490	1	m
	363	25.1	22.6	0	122 / 79	0[f]	3	/	33+6	36+4	19	s.c.	1905	3	f
	364	37.9	22.1	0	150 / 80	0[f]	0	/	40+3	40+3	0	s.c.	2665	2	m
	365	41.5	28.0	0	155/101	200	0	PE	30+2	32+0	12	s.c.	975	3	f

Supplemental Table 1 (continued)

	mothers											infants		
	patient / individual	age a)	BMI [kg/m^2]	parity a)	median blood pressure a.b) [mmHg]	proteinuria [mg/l] a.c.d)	smoking status e)	co-morb. f)	gestational age at collection of blood [w+d] g)	gestational age at delivery [w+d] g)	Δd h)	mode of delivery i)	birth weight [g]	birth weight percentile	sex
SGA I	201	17.7	21.8	0	109 / 64	69	0	/	34 + 4	37 + 5	22	vaginal	2300	3	m
	202	31.8	20.0	2	120 / 80	84	0	/	32 + 3	38 + 3	42	s.c.	2545	4	f
	203	27.2	28.1	0	114 / 60	0	0	/	29 + 5	36 + 1	45	s.c.	2000	3	m
	204	25.6	16.2	0	138 / 48	0	0	/	32 + 3	38 + 4	43	s.c.	2740	6	m
	205	31.0	25.8	0	146 / 84	0	15	/	31 + 4	37 + 5	43	s.c.	2455	5	f
	206	30.5	24.5	1	132 / 62	0	0	/	29 + 5	36 + 1	45	s.c.	2040	4	f
	207	21.4	25.0	0	104 / 59	70	5	/	32 + 1	40 + 5	60	vaginal	2780	3	f
	208	40.2	26.0	1	120 / 75	0	0	/	32 + 2	38 + 2	42	s.c.	2590	7	f
	209	36.6	21.9	1	84 / 45	0	0	/	33 + 6	37 + 3	25	s.c.	1950	1	m
	210	19.7	18.4	0	104 / 73	0	30	/	31 + 4	38 + 1	46	vaginal	2750	8	m
	211	36.1	25.5	5	121 / 73	0	0	/	32 + 6	38 + 3	39	vaginal	2560	3	m
	212	40.2	20.6	1	117 / 70	0	0	/	30 + 1	37 + 2	50	s.c.	2560	9	m
	213	27.0	21.1	0	110 / 70	0	0	/	22 + 6	37 + 6	105	s.c.	2170	2	f
	214	22.9	18.2	0	110 / 60	0	15	/	33 + 0	39 + 0	42	vaginal	2780	5	m
	215	25.0	23.6	0	134 / 71	0	0	/	25 + 6	40 + 1	100	s.c.	2750	4	f

a) determined at begining of hospitalization (time point of blood collection)
b) systolic/diastolic pressure
c) immunoturbidimetric assay (Tina-quant albumin; Roche Diagnostics. Mannheim, Germany); proteinuria is defined as urinary protein excretion >300 mg/L
d) dip stick assay; significant protein is present with readings of more than +1 (max: +3)
e) amount of cigarettes per day
f) preeclampsia
g) w: week, d: day
h) Δd: time difference between blood sample collection and delivery in days
i) s.c.: sectio caesarea
j) spectra not used for biostatistical analyses

Supplemental Table 2: Areas of ion signals selected for scoring.

clinical diagnosis	patient ID	measurement [a,b]	areas of ion signals (m/z)				
			apo CII [c)] 8205 [d)]	apo $CIII_0$ [c)] 8766 [d)]	pro-apo CII [c)] 8916 [d)]	apo $CIII_1$ [c)] 9422 [d)]	apo$CIII_2$ [c)] 9713 [d)]
CTRL	101	MS1	414.30	200.24	883.56	2074.71	539.74
CTRL	101	MS2	400.35	206.89	841.79	2383.00	622.14
CTRL	102	MS1	698.00	150.34	1247.64	1569.02	681.66
CTRL	102	MS2	705.71	133.29	1264.78	1534.71	652.39
CTRL	103	MS1	421.90	107.80	921.31	1840.63	402.83
CTRL	103	MS2	457.38	105.68	1019.10	1990.28	489.84
CTRL	104	MS1	264.59	116.04	961.72	2034.77	467.03
CTRL	104	MS2	178.14	106.47	658.71	1933.72	420.97
CTRL	105	MS1	500.00	156.60	977.96	2285.33	593.77
CTRL	105	MS2	464.62	141.43	920.55	2197.40	587.02
CTRL	106	MS1	222.37	126.91	791.88	1778.87	394.30
CTRL	106	MS2	213.55	143.11	763.30	1885.79	430.03
CTRL	107	MS1	438.96	159.81	970.72	2400.46	409.66
CTRL	107	MS2	475.35	127.37	939.19	2109.18	364.65
CTRL	108	MS1	377.30	232.00	727.85	2564.50	554.57
CTRL	108	MS2	383.90	203.30	684.85	2141.79	398.44
CTRL	109	MS1	329.15	106.16	911.18	1942.23	492.75
CTRL	109	MS2	325.66	108.23	917.82	1948.87	509.50
CTRL	110	MS1	390.37	281.28	982.85	1860.78	523.74
CTRL	110	MS2	380.13	265.66	907.92	1739.45	446.88
CTRL	111	MS1	238.20	102.48	886.59	1953.43	479.76
CTRL	111	MS2	251.44	101.71	936.77	2040.18	482.76
CTRL	112	MS1	312.80	238.63	967.43	2128.26	439.25
CTRL	112	MS2	324.25	234.88	974.10	2150.88	464.01
CTRL	113	MS1	254.58	278.50	1027.06	2026.04	427.75
CTRL	113	MS2	281.71	292.17	1106.54	2132.15	461.05
CTRL	114	MS1	144.47	182.59	589.61	1461.07	320.83
CTRL	114	MS2	152.05	178.65	584.65	1455.51	327.33
CTRL	115 [e)]	MS1	281.34	14.20	1167.01	1110.15	413.78
CTRL	115 [e)]	MS2	231.69	18.74	937.52	977.23	402.52
SGA	201	MS1	215.62	270.94	789.79	1330.84	339.23
SGA	201	MS2	263.98	460.72	1037.82	2435.07	655.13
SGA	202	MS1	732.59	93.49	876.16	2469.79	564.72
SGA	202	MS2	654.59	91.84	769.69	2215.84	510.94
SGA	203	MS1	233.77	141.40	1082.29	2776.67	526.12
SGA	203	MS2	233.77	141.40	1082.29	2776.67	526.12
SGA	204	MS1	306.25	262.94	937.48	2267.95	442.92
SGA	204	MS2	273.12	283.39	844.56	2332.37	435.84
SGA	205	MS1	292.18	456.29	907.95	2217.01	629.64
SGA	205	MS2	217.11	399.58	691.47	1921.16	582.48
SGA	206	MS1	411.65	128.55	977.60	1703.53	552.24
SGA	206	MS2	433.39	131.13	1017.58	1770.33	649.78
SGA	207	MS1	520.37	184.78	1227.13	2247.78	404.80
SGA	207	MS2	523.52	165.27	1260.87	2034.50	375.76
SGA	208	MS1	120.34	99.83	679.65	1608.17	595.25
SGA	208	MS2	118.43	99.81	704.81	1762.08	614.28
SGA	209	MS1	483.31	80.24	908.62	1778.71	437.86
SGA	209	MS2	504.72	76.05	897.84	1826.20	476.90

Supplemental Table 2 (continued)

clinical diagnosis	patient ID	measurement [a,b]	areas of ion signals (m/z)				
			apo CII [c)] 8205 [d)]	apo $CIII_0$ [c)] 8766 [d)]	pro-apo CII [c)] 8916 d)	apo $CIII_1$ [c)] 9422 [d)]	$apoCIII_2$ [c)] 9713 [d)]
SGA	210	MS1	197.06	132.67	787.89	2419.87	418.73
SGA	210	MS2	168.81	124.32	679.19	2021.89	317.82
SGA	211	MS1	201.32	157.10	892.48	1903.22	420.12
SGA	211	MS2	170.87	130.35	781.20	1517.58	330.60
SGA	212	MS1	239.09	225.91	637.04	1840.71	497.30
SGA	212	MS2	293.11	217.61	766.70	1913.29	524.41
SGA	213	MS1	319.36	281.63	1057.62	2544.11	496.54
SGA	213	MS2	243.79	227.11	829.42	2215.08	422.14
SGA	214	MS1	464.05	152.31	979.33	2000.95	519.80
SGA	214	MS2	540.68	150.61	1129.53	2173.92	608.80
SGA	215	MS1	107.24	122.08	636.23	1336.81	363.48
SGA	215	MS2	157.04	130.62	883.08	1777.3	520.11
IUGR	301	MS1	192.37	235.80	825.99	2279.91	404.18
IUGR	301	MS2	194.27	245.49	853.32	2481.24	441.21
IUGR	302	MS1	567.62	81.83	1174.89	2088.35	639.09
IUGR	302	MS2	659.08	102.29	1320.98	2364.86	635.10
IUGR	303	MS1	364.36	220.96	1245.24	2128.28	554.69
IUGR	303	MS2	352.85	225.31	1252.01	2323.04	618.43
IUGR	304	MS1	304.41	132.04	1112.63	1861.06	375.89
IUGR	304	MS2	263.48	123.54	940.25	1765.63	404.76
IUGR	305	MS1	217.45	163.06	1083.87	1687.21	452.45
IUGR	305	MS2	236.67	147.27	1180.20	1695.96	457.25
IUGR	306	MS1	220.31	122.83	1070.73	1806.31	430.47
IUGR	306	MS2	232.44	126.49	1215.18	1946.77	444.06
IUGR	307	MS1	213.23	399.97	1071.57	2112.83	424.41
IUGR	307	MS2	139.88	343.33	671.93	1850.29	389.32
IUGR	308	MS1	212.31	130.90	929.72	1175.19	592.87
IUGR	308	MS2	201.66	129.91	856.22	1109.21	598.61
IUGR	309	MS1	239.50	166.24	1135.39	1948.42	395.73
IUGR	309	MS2	233.65	134.17	1094.62	1505.65	308.34
IUGR	310	MS1	418.20	97.19	934.46	1417.34	383.97
IUGR	310	MS2	484.28	88.89	1051.94	1347.04	307.99
IUGR	311	MS1	262.66	99.87	937.06	1606.35	339.85
IUGR	311	MS2	263.28	99.37	958.19	1638.39	353.77
IUGR	312	MS1	477.13	145.12	1254.09	1709.73	469.29
IUGR	312	MS2	480.48	142.78	1233.48	1666.21	433.14
IUGR	313	MS1	180.27	183.17	1029.00	1663.82	331.00
IUGR	313	MS2	191.68	186.87	1156.24	1830.70	396.27
IUGR	314	MS1	293.84	105.08	924.29	1241.76	235.34
IUGR	314	MS2	434.38	124.61	1449.40	1512.45	269.20
IUGR	315 [e)]	MS1	n.d.	n.d.	n.d.	n.d.	n.d.
IUGR	315 [e)]	MS2	n.d.	n.d.	n.d.	n.d.	n.d.

a) MS1: first measurement of ClinProt work-up, (“training set”).
b) MS2: second measurement of ClinProt work-up, (“test set”).
c) abbreviated protein name
d) m/z value in MALDI mass spectrum
e) n.d.: ion signal areas not determined

Supplemental Table 3: Determination of Youden Index (Jmax) for Quotient A.

**patient ID**	**clinical diagnosis**	**quotient A** [a)]	**test cut-off**	**below cut-off**	**above cut-off**	**TP** [b)]	**FP** [b)]	**TN** [b)]	**FN** [b)]	**sensitivity**	**specificity**	**J** [c)]
	min	0.8										
102	CTRL	1.8	1.3	0	28	14	14	0	0	1.00	0.00	0.00
108	CTRL	1.9	1.9	1	27	14	13	1	0	1.00	0.07	0.07
105	CTRL	2.0	1.9	2	26	14	12	2	0	1.00	0.14	0.14
302	IUGR	2.1	2.0	3	25	14	11	3	0	1.00	0.21	0.21
101	CTRL	2.1	2.1	4	24	13	11	3	1	0.93	0.21	0.14
103	CTRL	2.2	2.2	5	23	13	10	4	1	0.93	0.29	0.21
107	CTRL	2.2	2.2	6	22	13	9	5	1	0.93	0.36	0.29
310	IUGR	2.2	2.2	7	21	13	8	6	1	0.93	0.43	0.36
110	CTRL	2.5	2.4	8	20	12	8	6	2	0.86	0.43	0.29
312	IUGR	2.6	2.6	9	19	12	7	7	2	0.86	0.50	0.36
109	CTRL	2.8	2.7	10	18	11	7	7	3	0.79	0.50	0.29
112	CTRL	3.1	2.9	11	17	11	6	8	3	0.79	0.57	0.36
314	IUGR	3.1	3.1	12	16	11	5	9	3	0.79	0.64	0.43
303	IUGR	3.4	3.3	13	15	10	5	9	4	0.71	0.64	0.36
106	CTRL	3.6	3.5	14	14	9	5	9	5	0.64	0.64	0.29
311	IUGR	3.6	3.6	15	13	9	4	10	5	0.64	0.71	0.36
104	CTRL	3.6	3.6	16	12	8	4	10	6	0.57	0.71	0.29
304	IUGR	3.7	3.6	17	11	8	3	11	6	0.57	0.79	0.36
111	CTRL	3.7	3.7	18	10	7	3	11	7	0.50	0.79	0.29
113	CTRL	4.0	3.9	19	9	7	2	12	7	0.50	0.86	0.36
114	CTRL	4.1	4.1	20	8	7	1	13	7	0.50	0.93	0.43
301	IUGR	4.3	**4.2**	**21**	**7**	**7**	**0**	**14**	**7**	**0.50**	**1.00**	**0.50**
308	IUGR	4.4	4.3	22	6	6	0	14	8	0.43	1.00	0.43
309	IUGR	4.7	4.6	23	5	5	0	14	9	0.36	1.00	0.36
306	IUGR	4.9	4.8	24	4	4	0	14	10	0.29	1.00	0.29
305	IUGR	5.0	4.9	25	3	3	0	14	11	0.21	1.00	0.21
307	IUGR	5.0	5.0	26	2	2	0	14	12	0.14	1.00	0.14
313	IUGR	5.7	5.4	27	1	1	0	14	13	0.07	1.00	0.07
	max	6.7	6.2	28	0	0	0	14	14	0.00	1.00	0.00

a) ranking according to the values of quotients A after adjoining theoretical minimum and maximum values

b) FN = false negative, FP = false positive, TN = true negative, TP = true positive.

c) J=Youden index; bold print: J_{max}

Supplemental Table 4: Determination of Youden Index (Jmax) for Quotient B.

patient ID	clinical diagnosis	quotient B [a)]	test cut-off	below cut-off	above cut-off	TP [b)]	FP [b)]	TN [b)]	FN [b)]	sensitivity	specificity	J [c)]
	min	2.0										
302	IUGR	3.0	2.5	0	28	14	14	0	0	1.00	0.00	0.00
111	CTRL	4.2	3.6	1	27	13	14	0	1	0.93	0.00	-0.07
109	CTRL	4.4	4.3	2	26	13	13	1	1	0.93	0.07	0.00
104	CTRL	4.6	4.5	3	25	13	12	2	1	0.93	0.14	0.07
103	CTRL	4.8	4.7	4	24	13	11	3	1	0.93	0.21	0.14
311	IUGR	5.1	**5.0**	**5**	**23**	**13**	**10**	**4**	**1**	**0.93**	**0.29**	**0.21**
310	IUGR	5.4	5.3	6	22	12	10	4	2	0.86	0.29	0.14
105	CTRL	5.4	5.4	7	21	11	10	4	3	0.79	0.29	0.07
306	IUGR	5.5	5.5	8	20	11	9	5	3	0.79	0.36	0.14
107	CTRL	5.7	5.6	9	19	10	9	5	4	0.71	0.36	0.07
106	CTRL	5.8	5.8	10	18	10	8	6	4	0.71	0.43	0.14
304	IUGR	5.9	5.9	11	17	10	7	7	4	0.71	0.50	0.21
312	IUGR	6.7	6.3	12	16	9	7	7	5	0.64	0.50	0.14
102	CTRL	6.7	6.7	13	15	8	7	7	6	0.57	0.50	0.07
309	IUGR	7.1	6.9	14	14	8	6	8	6	0.57	0.57	0.14
314	IUGR	7.1	7.1	15	13	7	6	8	7	0.50	0.57	0.07
308	IUGR	7.4	7.3	16	12	6	6	8	8	0.43	0.57	0.00
108	CTRL	7.4	7.4	17	11	5	6	8	9	0.36	0.57	-0.07
305	IUGR	7.6	7.5	18	10	5	5	9	9	0.36	0.64	0.00
101	CTRL	7.7	7.6	19	9	4	5	9	10	0.29	0.64	-0.07
303	IUGR	8.2	7.9	20	8	4	4	10	10	0.29	0.71	0.00
301	IUGR	8.8	8.5	21	7	3	4	10	11	0.21	0.71	-0.07
313	IUGR	9.2	9.0	22	6	2	4	10	12	0.14	0.71	-0.14
112	CTRL	9.3	9.2	23	5	1	4	10	13	0.07	0.71	-0.21
114	CTRL	10.2	9.8	24	4	1	3	11	13	0.07	0.79	-0.14
113	CTRL	11.3	10.8	25	3	1	2	12	13	0.07	0.86	-0.07
110	CTRL	11.8	11.6	26	2	1	1	13	13	0.07	0.93	0.00
307	IUGR	15.8	13.8	27	1	1	0	14	13	0.07	1.00	0.07
	max	16.8	16.3	28	0	0	0	14	14	0.00	1.00	0.00

a) ranking according to the values of quotients A after adjoining theoretical minimum and maximum values
b) FN = false negative, FP = false positive, TN = true negative, TP = true positive.
c) J=Youden index; bold print: J_{max}

Supplemental Table 5: Determination of Youden Index (Jmax) for Quotient C.

patient ID	clinical diagnosis	quotient C [a)]	test cut-off	below cut-off	above cut-off	TP [b)]	FP [b)]	TN [b)]	FN [b)]	sensitivity	specificity	J [c)]
	min	1.2										
108	CTRL	2.2	1.7	0	28	14	14	0	0	1.00	0.00	0.00
301	IUGR	2.8	2.5	1	27	14	13	1	0	1.00	0.07	0.07
114	CTRL	3.0	2.9	2	26	13	13	1	1	0.93	0.07	0.00
101	CTRL	3.1	3.1	3	25	13	12	2	1	0.93	0.14	0.07
105	CTRL	3.2	3.2	4	24	13	11	3	1	0.93	0.21	0.14
107	CTRL	3.3	3.3	5	23	13	10	4	1	0.93	0.29	0.21
106	CTRL	3.4	3.4	6	22	13	9	5	1	0.93	0.36	0.29
112	CTRL	3.4	3.5	7	21	13	8	6	1	0.93	0.43	0.36
111	CTRL	3.5	3.5	8	20	13	7	7	1	0.93	0.50	0.43
109	CTRL	3.6	3.5	9	19	13	6	8	1	0.93	0.57	0.50
307	IUGR	3.6	3.6	10	18	13	5	9	1	0.93	0.64	0.57
104	CTRL	3.7	3.7	11	17	12	5	9	2	0.86	0.64	0.50
110	CTRL	3.7	3.7	12	16	12	4	10	2	0.86	0.71	0.57
113	CTRL	3.8	3.7	13	15	12	3	11	2	0.86	0.79	0.64
103	CTRL	3.9	3.8	14	14	12	2	12	2	0.86	0.86	0.71
302	IUGR	4.2	**4.0**	**15**	**13**	**12**	**1**	**13**	**2**	**0.86**	**0.93**	**0.79**
303	IUGR	4.3	4.2	16	12	11	1	13	3	0.79	0.93	0.71
309	IUGR	4.5	4.4	17	11	10	1	13	4	0.71	0.93	0.64
306	IUGR	4.5	4.5	18	10	9	1	13	5	0.64	0.93	0.57
311	IUGR	4.6	4.6	19	9	8	1	13	6	0.57	0.93	0.50
304	IUGR	4.7	4.6	20	8	7	1	13	7	0.50	0.93	0.43
305	IUGR	4.7	4.7	21	7	6	1	13	8	0.43	0.93	0.36
313	IUGR	4.7	4.7	22	6	5	1	13	9	0.36	0.93	0.29
308	IUGR	4.9	4.8	23	5	4	1	13	10	0.29	0.93	0.21
310	IUGR	4.9	4.9	24	4	3	1	13	11	0.21	0.93	0.14
102	CTRL	5.2	5.1	25	3	2	1	13	12	0.14	0.93	0.07
312	IUGR	5.4	5.3	26	2	2	0	14	12	0.14	1.00	0.14
314	IUGR	5.8	5.6	27	1	1	0	14	13	0.07	1.00	0.07
	max	6.8	6.3	28	0	0	0	14	14	0.00	1.00	0.00

a) ranking according to the values of quotients A after adjoining theoretical minimum and maximum values

b) FN = false negative, FP = false positive, TN = true negative, TP = true positive.

c) J=Youden index; bold print: J_{max}

Supplemental Table 6: Power analysis of mass spectrometric ion signal-derived proteome profile series [a)].

no.	type of data set	group	mean	std dev.	required sample size	used sample size [b)]	type I error (α)	type II error (β)	actual power
1	**training "O": CTRL I vs FGR I**	CTRL	0.79	0.58	3	14	0.05	0.20	0.81
	(CTRL I, n=14; FGR I, n=14) [b)]	FGR	2.29	0.61	3	14			
2	**"development" test: CTRL I+II vs FGR I+II**	CTRL	0.91	0.75	9	28	0.05	0.20	0.83
	(CTRL I+II, n=28; FGR I+II, n=29) [c)]	FGR	1.82	0.67	9	44			
3	**"validation" test: CTRL I+II and SGA I vs FGR I+II**	CTRL+SGA	0.93	0.65	5	42	0.05	0.20	0.81
	(CTRL I+II, n=28; SGA I, n=14; FGR I+II, n=29) [c)]	FGR	2.15	0.63	5	29			

a) cumulative score separator: >1 = FGR; ≤ 1 = CTRL.
b) number of individuals
c) only via Jmax determined cut-off values: quotient A = 4.2; quotient B = 5.0; quotient C = 4.0.
d) combined cut-off values: quotient A = 4.2 and 3.4; quotient B = 5.0 and 7.0; quotient C = 4.0 and 5.1.
